# Antifungal Activity and Computational Study of Constituents from *Piper divaricatum* Essential Oil against *Fusarium* Infection in Black Pepper

**DOI:** 10.3390/molecules191117926

**Published:** 2014-11-04

**Authors:** Joyce Kelly R. da Silva, José Rogério A. Silva, Soelange B. Nascimento, Shirlley F. M. da Luz, Erisléia N. Meireles, Cláudio N. Alves, Alessandra R. Ramos, José Guilherme S. Maia

**Affiliations:** 1 Programa de Pós-Graduação em Biotecnologia, Instituto de Ciências Biológicas, Universidade Federal do Pará, Belém, PA 66075-110, Brazil; E-Mails: shirlleyfm@hotmail.com (S.F.M.L.); leia.nm@hotmail.com (E.N.M.); 2 Laboratório de Planejamento e Desenvolvimento de Fármacos, Instituto de Ciências Exatas e Naturais, Universidade Federal do Pará, Belém, PA 66075-110, Brazil; E-Mails: rogerio@ufpa.br (J.R.A.S.); soegen@hotmail.com (S.B.N.); nahum@ufpa.br (C.N.A.); 3 Instituto de Estudos em Saúde e Biológicas, Universidade Federal do Sul e Sudeste do Pará, Marabá, PA 68501-970, Brazil; E-Mail: alessandra.rezende@gmail.com; 4 Programa de Pós-Graduação em Química, Instituto de Ciências Exatas e Naturais, Universidade Federal do Pará, Belém, PA 66075-110, Brazil; E-Mail: gmaia@ufpa.br

**Keywords:** *Piper divaricatum*, essential oil composition, *Fusarium solani* f. sp. *piperis*, antifungal activity, β-glicosidase inhibition, molecular docking

## Abstract

*Fusarium* disease causes considerable losses in the cultivation of *Piper* *nigrum*, the black pepper used in the culinary world. Brazil was the largest producer of black pepper, but in recent years has lost this hegemony, with a significant reduction in its production, due to the ravages produced by the *Fusarium solani* f. sp. *piperis*, the fungus which causes this disease. Scientific research seeks new alternatives for the control and the existence of other *Piper* species in the Brazilian Amazon, resistant to disease, are being considered in this context. The main constituents of the oil of *Piper divaricatum* are methyleugenol (75.0%) and eugenol (10.0%). The oil and these two main constituents were tested individually at concentrations of 0.25 to 2.5 mg/mL against *F. solani* f. sp. *piperis*, exhibiting strong antifungal index, from 18.0% to 100.0%. The 3D structure of the β-glucosidase from *Fusarium solani* f. sp. *piperis*, obtained by homology modeling, was used for molecular docking and molecular electrostatic potential calculations in order to determine the binding energy of the natural substrates glucose, methyleugenol and eugenol. The results showed that β-glucosidase (Asp45, Arg113, Lys146, Tyr193, Asp225, Trp226 and Leu99) residues play an important role in the interactions that occur between the protein-substrate and the engenol and methyleugenol inhibitors, justifying the antifungal action of these two phenylpropenes against *Fusarium solani* f. sp. *piperis*.

## 1. Introduction

*Piper nigrum* L. (Piperaceae), commonly known as black pepper, is one of the most famous and oldest spices in the world, with culinary and food preservative uses [1]. In the last century, the Japanese immigrants introduced the culture in Brazil, with excellent edaphic and climatic adaptation, driving the country to occupy the first place in world production. The Brazilian national harvest reached 44 thousand tons in 2011, with about 90% produced in the Pará State [2]. However, in last fifty years, the crop has been undermined by *Fusarium solani* f. sp. *piperis*, a biotrophic phytopathogen that causes the *Fusarium* infection with vessels obstruction and reduction of the culture, bringing serious social and economic losses for the regional producers [3].

Control measures for the *Fusarium* infection were initiated with research on the resistant black pepper specimens against the phytopathogen. However, the low genetic variability and the homogeneous plantations have also led to the spread of the fungus. Studies with *Piper* species from the Amazon, which were previously infected with *F. solani* f. sp. *piperis*, have identified plants with different response levels to the disease. Tolerant species have been used as rootstock, but, later, these plants exhibit incompatibility, and they die in the fourth year after cultivation [4]. Another alternative use for the native *Piper* species is the employment of their secondary metabolites as a natural fungicide. *Piper* essential oils and extracts have a high metabolic diversity, as the terpenes, phenylpropanoids, amides, lignans, neolignans, steroids, pyrones, piperolides, chalcones and flavones, which were previously reported as having antifungal properties [5,6].

The black pepper essential oil is associated with the numerous applications in the food and pharmacological industries and cosmetics and home remedies [7]. The aroma and flavor of black pepper essential oils are attributed to the presence of terpene compounds such as α- and β-pinene, sabinene, myrcene, limonene, β-caryophyllene and camphene [8]. Oil composition variability has been observed, according the major and popular cultivars. For instance, β-caryophyllene was identified in the leaf oil of all cultivars while α- and β-pinene, sabinene, myrcene, and limonene were not detected [9,10]. However, these terpene constituents are not defined as compounds having antifungal activity.

Previously, it was seen that the essential oil of *Piper divaricatum* occurring in the Marajó Island, Pará State, Brazil, is rich in eugenol and methyleugenol, two phenylpropenes with potent antifungal activity [11]. Seedlings of *Piper divaricatum* introduced in a nursery containing plants of black pepper infected with *F. solani* f. sp. *piperis* showed complete resistance to attack of the fungus, indefinitely. It was suspected that the presence of methyleugenol and eugenol in the oil of *Piper divaricatum* inhibits this fungus infection. Based on this finding, it was considered important to investigate if this other *Piper* species could be used in the future for controlling the *Fusarium* infection, either as a rootstock or in protection of the soil spreading the dried leaves in the culture areas of black pepper.

Many advances have been made in the knowledge of pathogenic plant fungi, at the cellular and molecular level. The β-glucosidase proteins from fungi are attractive targets for the design of new antifungal agents to control the *Fusarium* disease in plants [12,13]. Thus, the aim of this work was to provide information on possible roles of the main compounds of the *P. divaricatum* oil in the mechanisms of action against *F. solani* f. sp. *piperis*, evaluate the *in vitro* antifungal assays and perform the study of 3D structure of the β-glucosidase of *F. solani* (*Fs*Bglc). The 3D study was based on the structure of a homologous β-glucosidase protein by applying the method of comparative modeling by homology. Additionally, the molecular docking and calculation of the molecular electrostatic potential were applied to analyze the details of the protein-ligand interactions between the natural substrate (glucose/glc) and the two main phenylpropanoids of *P. divaricatum*, in the complex with *Fs*Bglc.

## 2. Results and Discussion

### 2.1. Oil-Composition

The oil of aerial parts (leaves and thin stems) of *P. divaricatum* showed yield of 3.0%. GC-MS analysis identified 16 components and the major volatile constituents were the phenylpropenes methyleugenol (77.1%) eugenol (7.9%) and eugenol acetate (3.8%), comprising about 89% of the oil (Table 1), with similar result as previously *et al.* described [11]. Between the minor constituents of the oil, the monoterpenes α-pinene (2.1%) and β-pinene (3.2%) were the most representatives, totaling other 5.3% of the oil. Large amounts of phenylpropanoid compounds have been found in essential oils from *Piper* species occurring in the Brazilian Amazon: safrole in *P. hispidinervum* C. DC. (91.0%) and *P. callosum* Ruiz & Pav. (70.0%), 2,4-dimethoxy-1-propenylbenzene (46.0%) and 3,4-methylenedioxy-phenylpropan-1-one (40.0%) in *P. marginatum* Jacq. and dillapiole in *P. aduncum* L. (35%–95%) [14,15,16,17].

### 2.2. Antifungal Activity

The oil of *Piper divaricatum* showed high antifungal activity against *F. solani* f. sp. *piperis*, with inhibition above 90%. Methyleugenol and eugenol, the main constituents of *P. divaricatum* oil, were tested individually at concentrations between 0.25 and 2.5 mg/mL and they exhibited also strong antifungal activity against *F. solani* f. sp. *piperis*. The antifungal index for the oil of *P. divaricatum* ranged from 18.0% to 92.0%; the methyleugenol standard (95% pure) varied from 32.3% to 79.0%; and the eugenol standard (95% pure) inhibited totally the growth of the fungus, showing an antifungal index of 100%, at concentrations between 0.75 and 2.5 mg/mL. The MIC and IC_50_ values for the oil of *P. divaricatum* and the standards of methyleugenol and eugenol are shown in Table 2.

**Table 1:** Oil composition (%) of *P. divaricatum*.

Constituents	IR ^a^	IR ^b^	Oil ^c^ (%)
α- Pinene	942	932	2.1
β- Pinene	988	974	3.2
Limonene	1036	1024	1.1
*E*-β-Ocimene	1052	1044	0.8
Borneol	1178	1165	0.1
**Eugenol**	1361	1356	**7.9**
β-Elemene	1395	1389	0.1
**Metileugenol**	1419	1403	**77.1**
β-Gurjunene	1438	1431	0.3
α-Humulene	1462	1452	0.1
*trans*- Muurola-4(14),5-diene	1487	1493	1.4
Eugenol acetate	1524	1521	3.8
Elemicin	1553	1555	0.3
*E*-Nerolidol	1568	1561	0.1
Caryophyllene oxide	1586	1582	0.1
Monoterpene hydrocarbons			7.2
Oxygenated monoterpenes			0.1
Sesquiterpene hydrocarbons			1.9
oxygenated sesquiterpenes			0.2
phenylpropanoids			89.1
total			98.5

RI = Retention index (DB-5ms column); a = Calculated; b = Literature (Adams, 2007); c: Main compounds (>5%) highlighted in bold.

**Table 2:** Antifungal activity of the oil of *P. divaricatum* and the standards of eugenol and methyleugenol against *F. solani* f. sp. *piperis*.

Concentration (mg/mL)	Antifungal Index (%) *		
	*P. divaricatum* Oil	Methyleugenol	Eugenol
0.25	17.56 ± 0.00 ^a^	31.30 ± 13.48 ª	32.82 ± 16.57 ^a^
0.50	38.93 ± 4.77 ^b^	48.85 ± 11.88 ª	83.97 ± 15.02 ^b^
0.75	63.36 ± 0.00 ^c^	74.05 ± 8.64 ^b^	100.00 ± 0.00 ^b^
1.00	77.10 ± 10.49 ^d^	71.76 ± 2.16 ^b^	100.00 ± 0.00 ^b^
2.50	92.37 ± 3.50 ^d^	78.63 ± 2.86 ^b^	100.00 ± 0.00 ^b^
MIC	>2.50	>2.50	0.750
IC_50_	0.698	0.501	0.497

* Values are presented as mean ± SD; ^a–d^ Significant differences at *p* < 0.05 level.

The antifungal activity of the oil of *P. divaricatum* has been previously reported. It showed germination inhibition for the fungal plant pathogens *Marasmus perniciosa* [18], *Cladosporium cladosporioides* and *C. sphaerospermum* [11], at the equivalent action of the miconazole, used as standard antifungal compound. Some studies have considered the inhibitory properties of methyleugenol against phytopathogens. It has strongly inhibited the growth of *Alternaria humicola*, *Colletotrichum gloeosporioides*, *Rhizoctonia solani* and *Phytophthora cactorum*, showing the best activity against this latter microorganism, after promoting its structural hyphae damage [19,20]. The vapor of eugenol has fungicidal activity against *F. verticillioides*, a phytopathogen that infects maize kernels [21]. Some fungi that promote the wood decay, as *Lenzites betulina* and *Laetiporus sulphureus*, have strong susceptibility to the eugenol, with antifungal index of 100% [22]. Nevertheless, eugenol showed weak fungicidal activity against *Colletotrichum gloeosporioides*, *C. fragariae*, *C. acutatum* and *Botrytis cinerea*, tested by direct bioautography [19,23].

**Figure 1:**
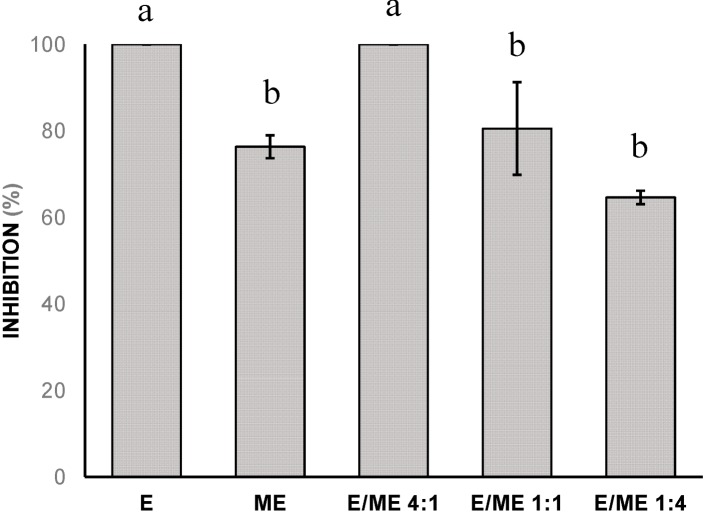
The antifungal index (%) of eugenol (E) and methyleugenol (ME) individually, and in combined effects of the proportions 4:1, 1:1 and 1:4, at concentration of 0.75 mg/mL, against *F. solani* f. sp. *piperis*. a, b Values with the same letter are not statistically different at the *p* < 0.05 level (Tukey’s test).

Combined effects of methyleugenol (ME) and eugenol (E) were found to determine whether this combination has synergistic, additive or antagonistic effects against *F. solani* f. sp *piperis*, as previously noted by Benz (1971) [24]. This interaction was evaluated by comparing the effects of the isolated methyleugenol and eugenol at concentration of 0.75 mg/mL, and in combination at proportions of 4:1, 1:1 and 1:4. The results are shown in Figure 1. The antifungal index for methyleugenol was 76.3% and for eugenol was 100%. The antifungal index for the combination 4ME/1E decreased to 64.6%, indicating an antagonist effect. For the combinations 1ME/1E and 1ME/4E with values of 80% and 100%, respectively, an additive effect was observed, and it was highest than the isolated effect of methyleugenol.

The significant antifungal activity of essential oil components arises from their hydrophobicity and could include attack on cell wall synthesis and retraction of cytoplasm in hyphae, which affects fungal growth and the morphogenesis, resulting in death of mycelium [24].

### 2.3. Homology Modeling and Others Structural Analysis

**Figure 2:**
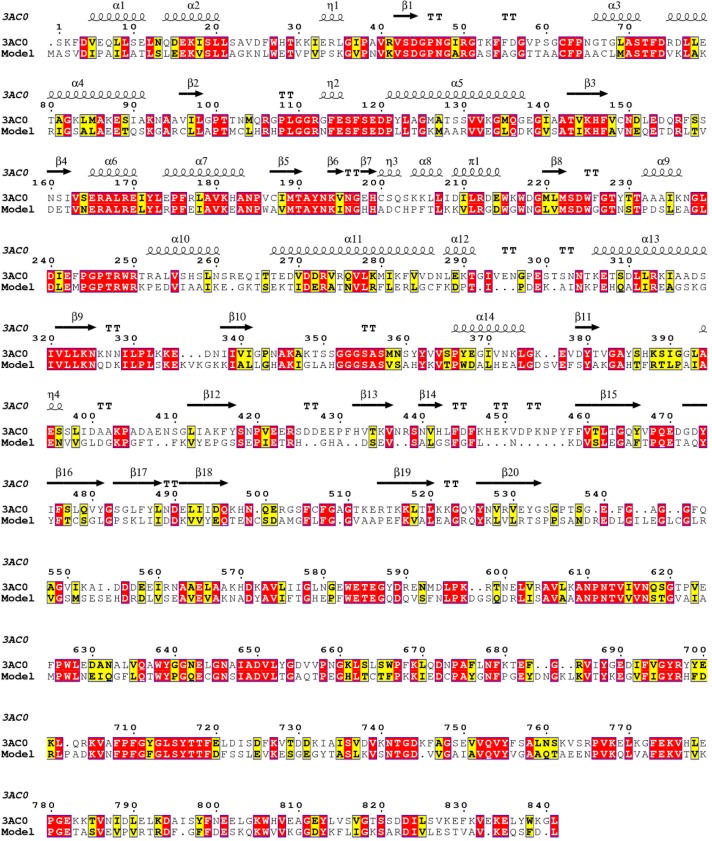
Primary sequential alignment for template (3AC0) and the target (Model): red for identical residues and yellow for similar residues.

**Figure 3:**
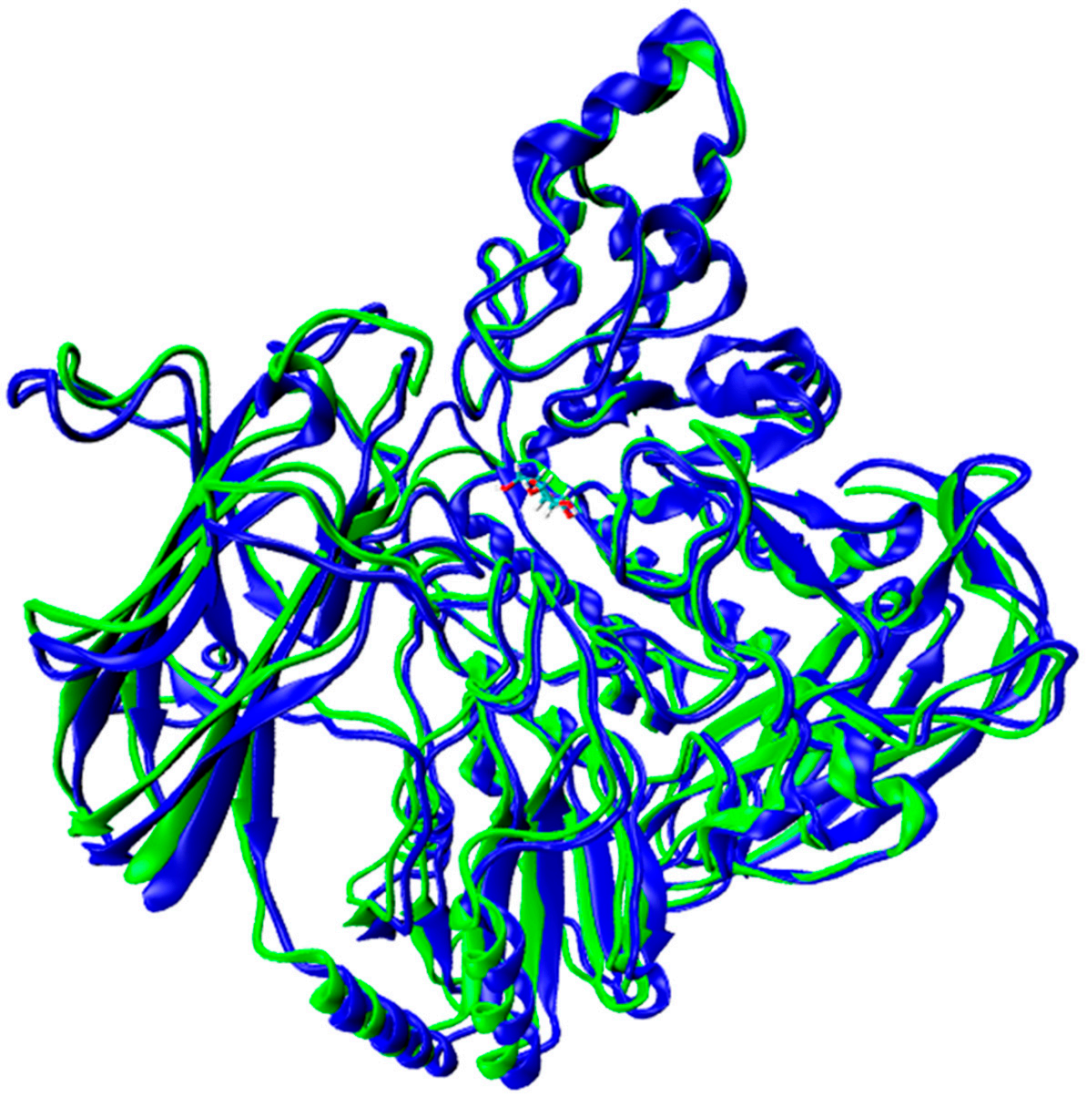
3D structural overlay for template (blue) and target (green).

As explained in the previous section, the target was obtained using the Swiss-Model Workspace server [25] and the MODELLER-9v12 program [26]. The enzyme β-glucosidase from *Kluyveromyces marxianus* (KmBglI) (PDB entry 3AC0) [27], in complex with glucose (glc), was found as template for *Fs*Bglc, where the target primary sequence has 38% of identity with template. A sequential alignment between template and target is shown in Figure 2. The superimposition of 3D structures of the β-glucosidases is shown in Figure 3 and, as can be seen, the 3D structures of the target and template are highly similar (RMSD = 0.249 Å). The main difference is founded in a loop localized between the β14 and β5 β-sheets of the tertiary structures, where a residual gap can be found (see Figure 2 and Figure 3). The results might be explained by the 3D model structure of *Fs*Bglc, only including the region corresponding to amino acids 1–340 in the amino-terminal, which is hypothesized for containing the catalytic domain. In addition, the amino acids residues Asp45, Leu99, Arg113, Lys146, His147, Met190, Tyr193, Asp225 and Trp226, which are normally present in the catalytic region, were conserved in the target structure. Others important residues close to the catalytic region, such as Phe445, Phe508 and Glu590, were also conserved, but with different number positions, Phe436, Phe491 and Glu576, respectively.

The stereochemical quality of the homologue model and template were checked with the PROCHECK program [28] as implemented in the Swiss-Model Workspace [25]. The majority of the residues of the Swiss-Model and template were found occupying the most favored regions of the Ramachandran plot while the other residues occupied the additionally allowed regions. These plots represent the distribution of the Φ and Ψ angles in each residual of the amino acid. In homologue model, 89.4% of the residues were presented in the most favored regions, 8.0% in the additional regions, 1.3% in the generously allowed regions and 1.3% in the disallowed regions. For template, these values are 86.5%, 12.9%, 0.4% and 0.1%, respectively. Furthermore, the quality of the structure derived from homology modeling was validated by calculating the QMEAN Z-score, a combined scoring function consisting of three potential statistical terms and two additional terms, describing the agreement of predicted and observed secondary structure and the solvent accessibility, respectively [29]. The Z-score value for the homologue model was −0.839 while the Z-score value for the template was −0.150. These results suggest that the structural quality of homologue model is better than template, model in some aspects.

**Figure 4:**
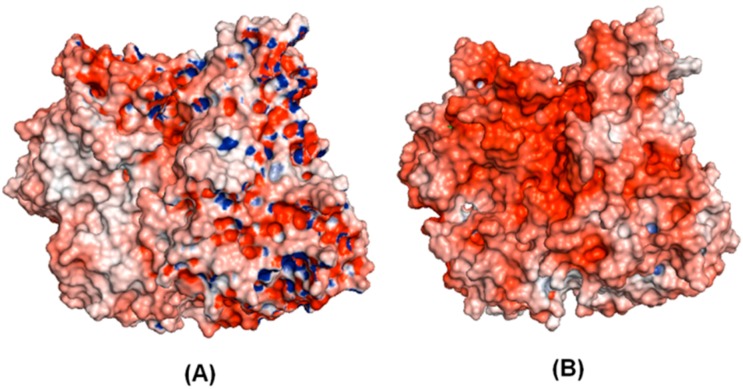
Electrostatic potential density surface obtained by Adaptive Poisson-Boltzmann Solver (APBS): template (**A**) and target (**B**).

The electrostatic potential density maps (see Figure 4) might be indicators of electrophilic and nucleophilic centers, which govern the strength of bonds, the strength of non-bonded interactions, and molecular reactivity. They affect the strength of the interaction of the ligand with the receptor protein. It is possible to see that target and template models have different electrostatic potential densities in some regions outside of protein. However, the region corresponding to amino acids 1–340 in the amino-terminal has similar electrostatic potential density. Therefore, according the residual identity, where the principal residues were conserved in homologue model.

### 2.4. Molecular Docking and MEP Analysis

**Figure 5:**
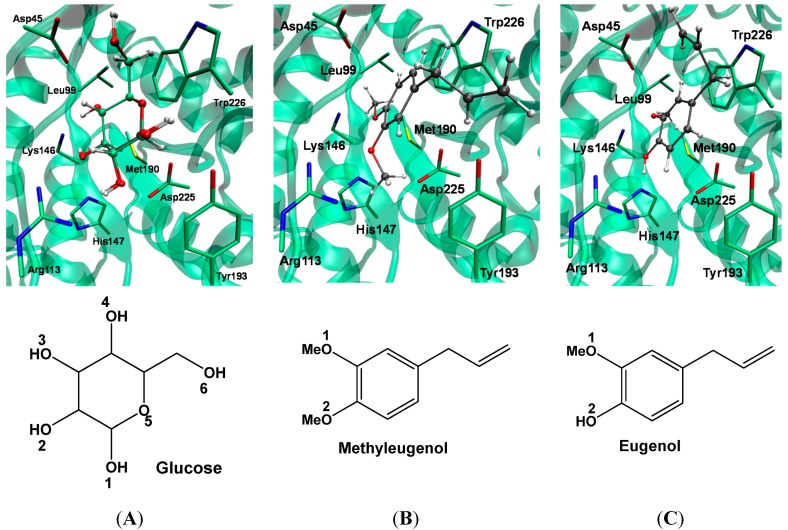
Re-docking conformational results for natural substrates: glucose (**A**); methyleugenol (**B**) and eugenol (**C**). The crystal reference is in gray carbon atoms, and theoretical result is in green carbon atoms. The residues are shown in tube model and the ligands in CPK model. The conformational results were obtained by molecular docking using MVD software.

A wide method of evaluating a docking program is to gauge its performance in cognate self-docking and re-docking. In this process, a ligand is removed from the crystal structure with its target protein and the program is challenged to pose the ligand so closely as possible to the experimental coordinates. Here, this methodology was applied to validate the MVD software, which was used in the structures of the β-glucosidases homologue protein. Then, the template and homologue protein were overlapped, and the glucose (glc) was docked in 3D structure obtained by homology modeling. The results are shown in Figure 5A, where the molecular docking is completely superimposed in the glc experimental conformation. The MVD software can reproduce the top conformation of β-glucosidase ligands inside the binding pocket of the FsBglc catalytic site. Furthermore, previous computational studies applying molecular docking in β-glucosidase homologue models have been used successfully [30]. The docking study was employed to determine the binding energy of the natural substrates (glucose/glc), eugenol and methyleugenol. The scoring results of docking calculations using the MolDock Score function and the H-bond interactions observed for glc and other ligands are summarized in Table 3.

Eugenol and methyleugenol were docked against the *Fs*Bglc homologue protein (see Figure 5B,C). The same docking procedure used in crystal ligand was applied for these compounds. The search algorithm MolDock SE, in combination with the MolDock scoring function and the H-bond interaction, produced the best docking results. In Table 3, it is possible to see that glucose has the lowest MolDock score and the H-bond energy. It reflects the affinity of the *Fs*Bglc homologue by natural substrate of β-glucosidase enzymes. The H-bond interactions of eugenol and methyleugenol show a better agreement with the glucose results, when compared with the MolDock score. It can be explained by various possibilities of hydrogen interactions formed between each ligand and the catalytic site, where residues as Asp45, Arg113, Lys146, Tyr193 and Asp225 are induced in forming hydrogen interactions with the polar groups of ligands, according the experimental results (Figure 4) [27]. Eugenol and methyleugenol can form 5 and 4 H-bond interactions, respectively (Table 4). The H-bond interactions are in agreement with the IC_50_ values shown in the previous section of experimental results (see Table 4), where eugenol and methyleugenol have similar inhibitory activity values. Furthermore, an interesting new interaction found for these compounds was the hydrogen interaction with the residue His147. This interaction cannot be found in complex with glucose.

**Table 3:** Energy calculations by MVD/MolDock Score and H-bond docking parameters.

Molecules	MolDock Score(kcal/mol)	H-Bond(kcal/mol)
Glucose	−106.45	−25.27
Eugenol	−83.89	−7.84
Methyleugenol	−68.16	−5.01

**Figure 6:**
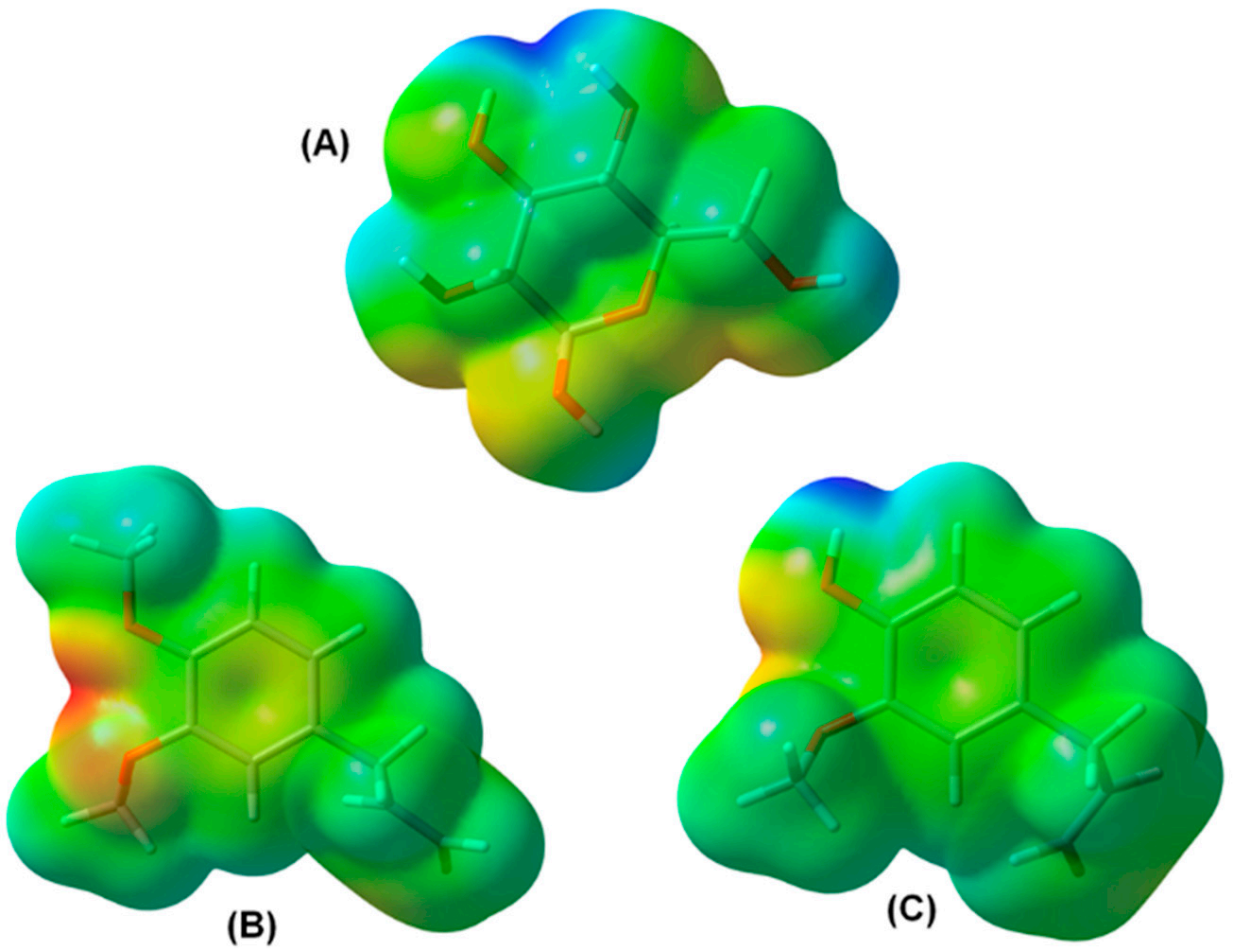
Molecular Electrostatic Potential (MEP) obtained by DFT theory using Gaussian-09 results: glucose (**A**); methyleugenol (**B**); and eugenol (**C**).

The nature of hydrogen bond interactions is still today the subject of many discussions and its importance in enzymatic environment has been shown in some drug design studies [31,32]. In this study, the hydrogen bond interactions were the main interactions found in protein-ligand complex as previously discussed. Thus, in order to understand why the eugenol and methyleugenol have showed similar interaction with the natural substrate, the molecular electrostatic potential (MEP) was built. MEP is a useful visual tool to understand the relative polarity of a molecule and serves to explain hydrogen bonding, reactivity, residual interaction, polarizability and the structure–activity relationship for biomolecules and drugs [32,33]. MEP was derived from DFT (B3LYP/6-31G*) calculations using Gaussian 09 package and visualized in Gaussview 3.07. These surfaces have an isodensity value of 0.002 a.u. In Figure 6, the most nucleophilic regions (negative electronic potential) are shown in red while the most electrophilic regions (positive electrostatic capability) have green color.

**Table 4:** Hydrogen bond interactions selected by H-bond score.

Molecules	Interactions	Molecule Atom	Receptor Atom	Distance (Å)
Glucose	8	O1	OH(Tyr193)	3.44
		O2	OD2(Asp225)	2.92
		O2	NH1(Arg113)	2.82
		O3	NH1(Arg113)	2.79
		O3	NZ(Lys146)	2.29
		O4	NZ(Lys146)	2.64
		O4	OD1(Asp45)	2.75
		O6	OD2(Asp45)	2.98
Eugenol	5	O1	OD1(Asp225)	3.12
		O1	NZ(Lys146)	2.33
		O2	NH2(Arg113)	2.87
		O2	NZ(Lys146)	2.92
		O2	NE2(His147)	2.52
Methyleugenol	4	O1	NH1(Arg113)	3.01
		O1	NH2(Arg113)	2.85
		O1	OD1(Asp225)	3.60
		O2	NZ(Lys146)	2.35

The natural substrate (glc) has an electronegative profile distributed around the whole molecule, this feature is important for the substrate be recognized by polar residues present in protein catalytic site, such as the residues Asp45, Arg113, Lys146, Tyr193 and Asp225. It can be observed the hydrogen bond interactions between OH groups of glc and the side chains of these amino acid residues (see Figure 6 and Table 3). Although eugenol and methyleugenol have fewer polar groups than glc, these compounds have shown a similar charge profile. This characteristic allows to seeing the same hydrogen bond interactions found in *Fs*Bglc-glc complex, where the phenyl group contributes to maintaining a similar electron density, as it was found in glc. A particular hydrophobic interaction can be found in all *Fs*Bglc-eugenol or *Fs*Bglc-methyleugenol complexes, where the aliphatic groups in these compounds interact with the side chain of residue Trp226 and the methoxy group (via methyl) interacts with the side chain of residue Leu99. It is in accordance with the hydrophobic properties found for these compounds in the experimental assays [24].

## 3. Experimental Section

### 3.1. Plant Material

The leaves and thin stems (aerial parts) of *Piper divaricatum* G. Meyer were collected in the Marajó Island, municipality of Breves, Pará State, Brazil. The species was identified by Dr Elsie F. Guimarães, Jardim Botânico do Rio de Janeiro, Rio de Janeiro, Brazil, and a voucher (MG 165212) was deposited in the herbarium of Emílio Goeldi Museum, city of Belém, Pará State, Brazil.

### 3.2. Extraction and Analysis of Essential Oils

The aerial parts (100 g) were subjected to hydrodistillation using a Clevenger-type apparatus (3 h). The extracted oil was dried over anhydrous sodium sulfate, and its percentage content was calculated on the basis of the plant dry weight. The analysis of the oil was carried out on a THERMO DSQ II GC–MS instrument, under the following conditions: DB-5ms (30 m × 0.25 mm; 0.25 mm film thickness) fused-silica capillary column; programmed temperature: 60–240 °C (3 °C/min); injector temperature: 250 °C; carrier gas: helium, adjusted to a linear velocity of 32 cm/s (measured at 100 °C); injection type: splitless (2 mL of a 1:1000 hexane solution); split flow was adjusted to yield a 20:1 ratio; septum sweep was a constant 10 mL/min; EIMS: electron energy, 70 eV; temperature of ion source and connection parts: 200 °C. The quantitative data regarding the volatile constituents were obtained by peak-area normalization using a FOCUS GC/FID operated under conditions similar the GC–MS, except for the carrier gas, which was nitrogen. The retention index was calculated for all the volatiles constituents using an n-alkane (C8–C40, Sigma-Aldrich, St. Louis, MO, USA) homologous series. Individual components were identified by comparison of both mass spectrum and GC retention data with authentic compounds which were previously analyzed and stored in private library, as well as with the aid of commercial libraries containing retention indices and mass spectra of volatile compounds commonly found in essential oils [34]. The standards of eugenol and methyleugenol were purchased from Sigma-Aldrich, Brazil.

### 3.3. Antifungal Assay

#### 3.3.1. Fungal Strains and Culture Media

The phytopathogen *Fusarium solani* f. sp. *piperis* was obtained from an infected plantation of *Piper nigrum* in Pará State, Brazil. The fungus was cultured on potato dextrose agar (PDA) for ten days, at 27 °C, before the experiment to be carried out.

#### 3.3.2. Effect of Essential oil on Mycelial Growth

The antifungal activity was performed by the agar dilution method [35] and expressed as percentage inhibition against the mycelia growth diameter. Aliquots of essential oil were incorporated in medium potato dextrose agar (PDA) sterilized, melting at concentration of 5 mg/mL. Mycelial discs (Ø, 0.9 cm) from *Fusarium solani* f. sp. *piperis* were transferred to the center of the plates and incubated in dark, at 27 °C. The mycelium growth was monitored during 7 days and the antifungal index was calculated according the Equation:

*Inhibition* (%) = [1 − (∅growth of treatment)/(∅growth of control)] × 100

All treatments were tested in quadruplicate, and plates without the oil were used as control.

#### 3.3.3. Determination of Minimum Inhibitory Concentration (MIC) and IC_50_

The oil of *Piper divaricatum*, eugenol and methyeugenol were incorporated in medium PDA at concentrations of 0.1, 0.25, 0.5, 0.75, 1.0 and 2.5 mg/mL. The minimum inhibitory concentration was defined as the lowest concentration able to cause 100% inhibition of mycelial growth. The IC_50_ values (concentration that inhibited 50% of the mycelium of fungi growth) were calculated by Probit analysis and nonlinear regression using the software GraphPad Prism 5.0.

#### 3.3.4. Statistical Analysis

Antifungal experiments were performed in quadruplicate and data analyzed are mean ± SE subjected to one-way ANOVA. Means are separated by Tukey’s multiple range tests when ANOVA was significant (*p* < 0.05) (GraphPad Prism 5.0).

### 3.4. Computational Studies

#### 3.4.1. Homology Modeling

Homology modeling is a well-known computational tool, which it is possible to build the secondary structure of a given protein based on knowledge of the primary structure [36]. This method is used to build protein structure, on the premise that specific functions are maintained during the evolutionary process between the conserved structurally homologous proteins [37]. As an initial step, the primary sequence of *F. solani* (NCBI code EEU48841.1) was obtained from the Genome Project, *Nectria haematococca* [38]. This sequence was subjected to BLAST search, in the Protein Data Bank (PDB), to identify suitable template structures for homology modeling. As homology modeling relies on sequence alignment, the target-template sequences were aligned using the Swiss-Model Workspace [25] and, after a careful examination of alignment errors, the automated comparative protein modeling program, MODELLER-9v12, was used to build the model [26]. The *β*-glucosidase from *Fusarium solani* (*Fs*Bglc) structure was analyzed and evaluated using PROCHECK program and QMEAN6 Z-score as implemented in Swiss-Model Workspace.

#### 3.4.2. Molecular Docking

The 3D structure of *Fs*Bglc obtained by homology modeling was used as start point for molecular docking calculations. In order to validate the methodology used by Molegro Virtual Docker (MVD) software [39], a re-docking was performed using as reference the glucose (glc) coordinates, in complex with the crystal structure of *Kluyveromyces marxianus* (*Km*BglI) [27]. Then, the structures of eugenol and methyleugenol were tested *in silico* procedure. The MolDock SE (Simplex Evolution) search algorithm was used in the docking studies as implemented in MVD software. Furthermore, the MolDockScore, which is an adaptation of the Differential Evolution (DE) algorithm, and the H-Bond interactions were used as scoring function. The MolDock Score function was used because it yields a higher docking accuracy than other state-of-art docking algorithms (MVD: 87%, Glide: 82%, Surflex: 75%, FlexX: 58%) [39]. For the pose generation, 1500 maximum interactions were used to selecting a population size of 50. These poses can generate tests for different torsion angles, rotations and translations, which evaluate the affected part of the molecule and chooses the value that results in the lowest energy contributions. The MolDockScore function (Escore) is defined by the following energy terms:

E*_score_* = E*_inter_* + E*_intra_*

where E*_inter_* is the ligand-protein interaction energy and E*_intra_* is the internal energy of the ligand. The E*_inter_* is determined by follow Equation:

$$E_{inter}=\underset{i=ligand}{\sum }\underset{j=protein}{\sum }\left[E_{PLP}\left(r_{ij}\right)+332.0\frac{q_{i}q_{j}}{4r_{ij}^{2}}\right]$$

The E_PLP_ term is a piecewise linear potential using two different sets of parameters: one for approximating the steric term (van der Waals) between atoms and the other stronger potential for hydrogen bonds. The E*_intra_* is calculated by follow Equation:

$$E_{intra}=\underset{i=ligand}{\sum }\underset{j=protein}{\sum }\left[E_{PLP}\left(r_{ij}\right)\right]+\underset{flexiblebonds}{\sum }A\left[1-cos\left(m\theta -\theta_{0}\right)\right]+E_{clash}$$

The double summation calculates all the energy terms involving pairs of atoms of the ligand, except those connected by two bonds. The second summation calculates the torsional energy, where θ is the torsional angles of the bond. The average of the torsional energy term contributions is used if several torsions can be determined. The last term, *E*clash, assigns a penalty of 1000, if the distance between two atoms (more than two bonds apart) is less than 2.0 Å. Thus, the E*_clash_* term punishes infeasible ligand conformations [39].

#### 3.4.3. Other Structural Analysis

Structural alignment of structures (template and target) and the electrostatic potential around the template and the modeled molecular systems were calculated by solving the nonlinear Poisson-Boltzmann equation, using finite difference method as implemented in Adaptive Poisson-Boltzmann Solver (APBS). The three-dimensional surfaces of the molecular electrostatic potential (MEP) were generated using the software Chimera and APBS [40], with an ionic strength of 12 mM. Mapping of the electrostatic potential onto the molecular surface of the protein was performed with a potential range from −10 eV to 10 eV. These calculations were carried using a PB2PQR Server [41]. After the docking calculation, the MEP was calculated using Gaussian-09 package, applying DFT (B3LYP/6-31G*) theory quantum level with CHELPG charges in vacuum, to verify the charge distribution profile for each molecule analyzed by molecular docking.

## 4. Conclusions

Methyleugenol and eugenol, the main constituents of the *P. divaricatum* oil, are responsible by the significant antifungal activity observed against *Fusarium solani*f. sp. *piperis*, which causes severe infection in black pepper. The interaction of *Fs*Bglc protein with the phenylpropanoid inhibitors is in agreement with the experimental data and, therefore, can provide valuable structural information for advances in rational drug design. The model also showed that the residues Leu99 and Trp226 can interact with eugenol and methyleugenol by hydrophobic interactions and, in this way justifying their antifungal action.

## References

[1] Nair K.P.P. (2011). Agronomy and Economy of Black Pepper and Cardamom: The “King” and “Queen” of Spices.

[2] Lemos O.F., Poltronieri M.C., Rodrigues S.M., Menezes I.C., Mondin M. (2011). Conservação e Melhoramento Genético da Pimenteira-do-reino (Piper nigrum L.) Associado às Técnicas de Biotecnologia.

[3] Duarte M.L.R., Albuquerque F.C., Duarte M.L.R. (1999). Doenças da cultura da pimenta-do-reino. Doenças de Plantas no Trópico Úmido.

[4] Albuquerque F.C., Duarte M.L.R., Benchimol R.L., Endo T. (2001). Resistência de piperáceas nativas da Amazônia à infecção causada por *Nectria haematococca* f. sp. *piperis*. Acta Amaz..

[5] Danelutte A.P., Costantin M.B., Delgado G.E., Braz-Filho R., Kato M.J. (2005). Divergence of secondary metabolism in cell suspension cultures and differentiated plants of *Piper cernuum* and *P. crassinervium*. J. Braz. Chem. Soc..

[6] Lago J.H.G., Chen A., Young M.C.M., Guimarães E.F., de Oliveira A., Kato M.J. (2009). Prenylated benzoic acid derivatives from *Piper aduncum* L. and *P. hostmannianum* C. DC. (Piperaceae). Phytochem. Lett..

[7] Ravindran P.N., Kallupurackal J.A., Peter P.K. (2004). Black pepper. Handbook of Herbs and Spices.

[8] Gopalakrishnan M., Padmakumari K.P., Jayalekshmy A., Narayanan C.S. (1993). Gas chromatographic analysis and odor profiles of few Indian genotypes of *Piper nigrum* L.. J. Essent. Oil Res..

[9] Zachariah T.J., Parthasarathy V.A., Parthasarathy V.A., Chempakam B., Zachariah T.J. (2008). Black pepper. Chemistry of Spices.

[10] Zachariah T.J., Safeer A.L., Jayarajan K., Leela N.K., Vipin T.M., Saji K.V., Shiva K.N., Parthasarathy V.A., Mammootty K.P. (2010). Correlation of metabolites in the leaf and berries of selected black pepper varieties. Sci. Hortic..

[11] Silva J.K.R., Andrade E.H.A., Guimarães E.F., Maia J.G.S. (2010). Essential oil composition, antioxidant capacity and antifungal activity of *Piper divaricatum*. Nat. Prod. Commun..

[12] Nascimento S.B., Cascardo J.C.M., Menezes I.C., Duarte M.L.R., Darnet S.H., Harada M.L., de Souza C.R.B. (2009). Identifying sequences potentially related to resistance response of *Piper tuberculatum* to *Fusarium solani* f. sp. *piperis* by suppression subtractive hybridization. Protein Pept. Lett..

[13] De Souza C.R.B., Brígida A.B.S., dos Santos R.C., Costa C.N.M., Darnet S.H., Harada M.L. (2011). Identification of sequences expressed during compatible Black pepper-*Fusarium solani* f. sp. *peperis* interaction. Acta Physiol. Plant..

[14] Maia J.G.S., Silva M.H.L., Luz A.I.R., Zoghbi M.G.B., Ramos L.S. (1987). Espécies de *Piper* da Amazônia ricas em safrol. Quim. Nova.

[15] Maia J.G.S., Zoghbi M.G.B., Andrade E.H.A., Santos A.S., Silva M.H.L., Luz A.I.R., Bastos C.N. (1998). Constituents of the essential oil of *Piper aduncum* L. growing wild in the Amazon region. Flavour Fragr. J..

[16] Andrade E.H.A., Carreira L.M.M., Silva M.H.L., Silva J.D., Bastos C.N., Sousa P.J.C., Guimarães E.F., Maia J.G.S. (2008). Variability in essential oil composition of *Piper marginatum* sensu lato. Chem. Biodivers..

[17] Almeida R.R.P., Souto R.N.P., Bastos C.N., Silva M.H.L., Maia J.G.S. (2009). Chemical variation in *Piper aduncum* and biological properties of its dillapiole rich essential oil. Chem. Biodivers..

[18] Silva D.M.M.H., Bastos C.N. (2007). Atividade antifúngica de óleos essenciais de espécies de *Piper* sobre *Crinipellis perniciosa*, *Phytophthora palmivora* e *Phytophthora capsici*. Fitopatol. Bras..

[19] Meepagala K.M., Sturtz G., Wedge D.E. (2002). Antifungal constituents of the essential oil fraction of *Artemisia dracunculus* L. var. *dracunculus*. J. Agric. Food Chem..

[20] Dan Y., Liu H.-Y., Gao W.-W., Chen S.-L. (2010). Activities of essential oils from *Asarum heterotropoides* var. *mandshuricum* against five phytopathogens. Crop Prot..

[21] Menniti A.M., Gregori R., Neri F. (2010). Activity of natural compounds on *Fusarium verticillioides* and fumonisin production in stored maize kernels. Int. J. Food Microbiol..

[22] Yen T.-B., Chang S.-T. (2008). Synergistic effects of cinnamaldehyde in combination with eugenol against wood decay fungi. Bioresour. Technol..

[23] Burt S. (2004). Essential oils: Their antibacterial properties and potential applications in foods—A review. Int. J. Food Microbiol..

[24] Benz G., Burges H.D., Hussey N.W. (1971). Synergism of micro-organisms and chemical inseticides. Microbial Control of Insects and Mites.

[25] Arnold K., Bordoli L., Kopp J., Schwede T. (2006). The SWISS-MODEL workspace: A web-based environment for protein structure homology modeling. Bioinformatics.

[26] Sali A., Blundell T.L. (1993). Comparative protein modeling by satisfaction of spatial restraints. J. Mol. Biol..

[27] Yoshida E., Hidaka M., Fushinobu S., Koyanagi T., Minami H., Tamaki H., Kitaoka M., Katayama T., Kumagai H. (2010). Role of a PA14 domain in determining substrate specificity of a glycoside hydrolase family 3 β-glucosidase from *Kluyveromyces marxianus*. Biochem. J..

[28] Laskowski R.A., Arthur M.W., Moss D.S., Thorton J.R. (1993). PROCHECK: A program to check the stereochemical quality of protein structures. J. Appl. Crystallogr..

[29] Benkert P., Biasini M., Schwede T. (2011). Toward the estimation of the absolute quality of individual protein structure models. Bioinformatics.

[30] Alencar N.A.N., de Sousa P.R.M., Silva J.R.A., Lameira J., Alves C.N., Martí S., Moliner V. (2012). Computational analysis of human OGA structure in complex with PUGNAc and NAG-Thiazoline derivatives. J. Chem. Inf. Model..

[31] Rozas I. (2007). On the nature of hydrogen bonds: An overview on computational studies and a word about patterns. Phys. Chem. Chem. Phys..

[32] Silva J.R.A., Lameira J., Alves C.N. (2012). Insights for design of *Tripanosoma cruzi* GAPDH inhibitors: A QM/MM MD of 1,3-bisphospho-D-glyceric acid analogs. Int. J. Quantum Chem..

[33] Nascimento J.P., Silva J.R.A., Lameira J., Alves C.N. (2013). Metal-dependent inhibition of HIV-1 integrase by 5CITEP inhibitor: A theoretical QM/MM approach. Chem. Phys. Lett..

[34] Adams R.P. (2007). Identification of Essencial Oil Components by Gas Chromatography/Mass Spectrometry.

[35] Taira S., Tawata S., Kobamoto N., Toyama S., Yasuda M. (1994). Synthesis and fungicidal activity of new 1,3,2-oxazaphospholidine2-sulfides. J. Pestic. Sci..

[36] Martí-Renom M.A., Stuart A.C., Fiser A., Sánchez R., Melo F., Sali A. (2000). Comparative protein structure modeling of genes and genomes. Annu. Rev. Biophys. Biomol. Struct..

[37] Moraes G., Azevedo V., Costa M., Miyoshi A., Silva A., da Silva V., de Oliveira D., Teixeira M.F., Lameira J., Alves C.N. (2012). Homology modeling, molecular dynamics and QM/MM study of the regulatory protein PhoP from *Corynebacterium pseudotuberculosis*. J. Mol. Model..

[38] Coleman J.J., Rounsley S.D., Rodriguez-Carres M., Kuo A., Wasmann C.C., Grimwood J., Schmutz J., Taga M., White G.J., Zhou S. (2009). The Genome of *Nectria haematococca*: Contribution of supernumerary chromosomes to gene expansion. PLoS Genet..

[39] Thomsen R., Christensen M. (2006). MolDock: A new technique for high-accuracy molecular docking. J. Med. Chem..

[40] Holst M., Baker N., Wang F. (2000). Adaptive multilevel finite element solution of the Poisson-Boltzmann equation I. Algorithms and examples. J. Comput. Chem..

[41] Dolinsky T.J., Nielsen J., McCammon J., Baker N.A. (2004). PDB2PQR: An automated pipeline for the setup, execution, and analysis of Poisson-Boltzmann electrostatics calculations. Nucleic Acids Res..

